# Ameba-associated Keratitis, France

**DOI:** 10.3201/eid1707.100826

**Published:** 2011-07

**Authors:** Gaëlle Cohen, Louis Hoffart, Bernard La Scola, Didier Raoult, Michel Drancourt

**Affiliations:** Author affiliations: Hôpital de la Timone, Marseille, France (G. Cohen, L. Hoffart);; Université de la Méditerranée, Marseille (B. La Scola, D. Raoult, M. Drancourt)

**Keywords:** keratitis, ameba, contact lenses, Mycobacterium chelonae, viruses, mimivirus, virophage, Babela massiliensis, France, letter

**To the Editor:** Amebic keratitis is an aggressive infection usually associated with soft contact lenses, and its poor outcome can lead to a corneal graft (*1*). Ameba can host ameba-resistant bacteria (*2*) and serve as a source for numerous organisms to exchange DNA, adapt to changing environments, and become pathogenic to the host (*3*). However, mixed keratitis caused by amebae in association with ameba-resistant pathogens is rarely seen.

A 17-year-old woman who was myopic and had worn soft contact lenses for 3 years consulted our ophthalmology department for pain and redness of the left eye that had persisted for 2 weeks. No visual loss was reported, and a slit-lamp examination showed a millimetric epithelial defect associated with a round stromal infiltrate (Figure). No intraocular reaction was observed, and bacterial keratitis was diagnosed. Her condition improved after a 7-day topical fluoroquinolone treatment, and follow-up at 13 months showed only a slight superficial stromal scar. The patient reported that she had inappropriately worn monthly contact lenses for 3 months, cleaned and rinsed lenses with a commercial cleaning solution but diluted the solution with tap water, and washed her hands with tap water but did not dry them before handling the lenses.

**Figure:**
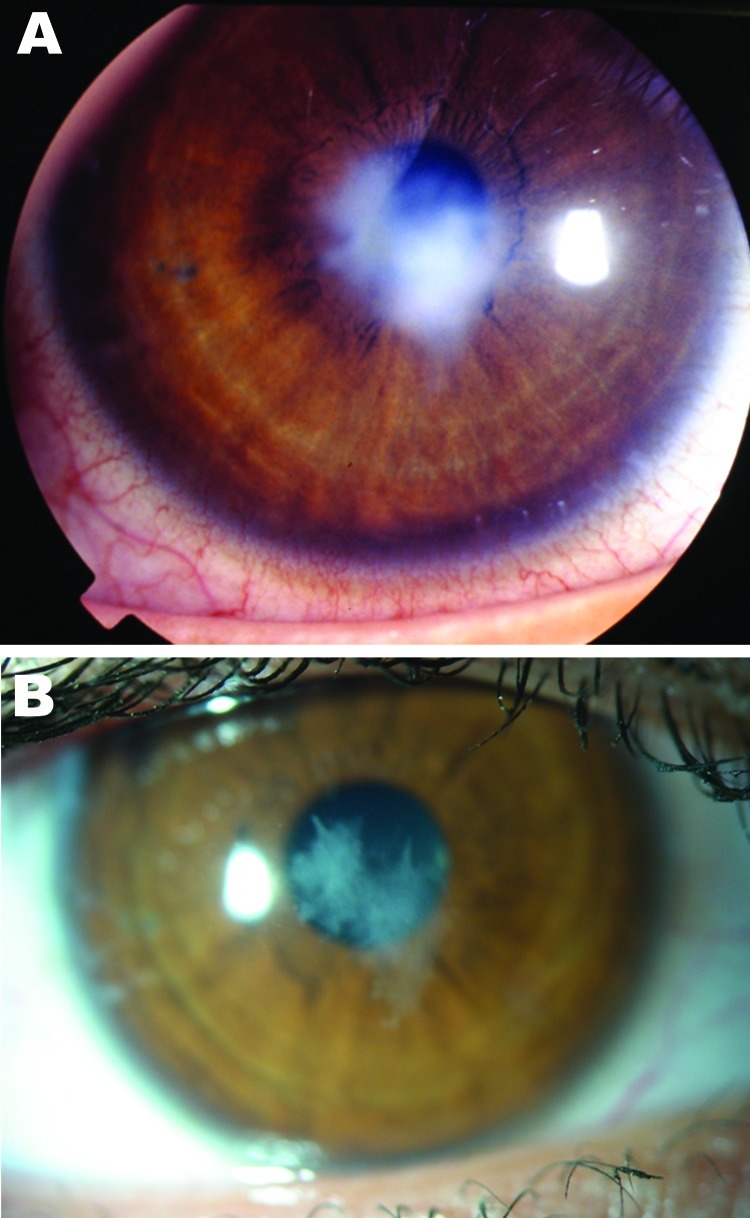
Ameba-associated keratitis in a 17-year-old woman (contact lens wearer), France, showing a paracentral corneal scar (A) and recovery at 13-month follow-up (B). Original magnification ×10.

Results of microbiologic analysis of a corneal scraping, which included molecular detection of ameba (18S rDNA), bacteria (16S rDNA), and herpesvirus (DNA polymerase), were negative. However, culture of contact lens storage case liquid in Page-modified Neff ameba saline enriched with heat-killed *Enterobacter aerogenes* (*2*) identified *Pseudomonas fluorescens* and *Stenotrophomonas maltophilia*, both of which were identified by matrix-assisted laser desorption ionization time-of-flight mass spectrometry (*4*); *Mycobacterium chelonae*, which was identified by *rpo*B gene sequencing; and *Acanthamoeba polyphaga*, which was identified by partial 18S rDNA sequencing.

Culturing this ameba in sterile peptone–yeast extract–glucose broth showed that it hosted 4 organisms. The first organism was a new intracellular Deltaproteobacterium provisionally named *Candidatus* Babela massiliensis (100% 16S rDNA sequence similarity with a reference strain [GenBank accession no. GQ495224], which was susceptible to 200 μg/mL rifampin and resistant to 20 μg/mL ciprofloxacin). The second organism was a new, unnamed, gram-negative, ciprofloxacin-susceptible (20 μg/mL) *Alphaproteobacterium* bacillus with 99% 16S rDNA sequence similarity (GenBank accession no. HM138368) with *Candidatus* Odyssella sp. and *Acanthamoeba* endosymbiont KA/E9 (*5*). The third organism was a new giant virus related to an *A*. *polyphaga* mimivirus (*6*) strain Lentille. The fourth organism was a new virophage referred to as Sputnik 2 (*7*).

*Acanthamoeba* spp. are ubiquitous in tap water (*2**,**5*), including that used by contact lens wearers to wash hands before manipulating the lens (*8*). Tap water hosts many organisms, including bacteria (*2**,**5*); large DNA viruses (*9*); and the recently described Sputnik virophage, a virus that infects ameba-resistant large DNA viruses (*10*). We found 5 bacterial species, a giant virus, and a virophage in the contact lens storage case liquid for this patient.

Although *Acanthamoeba* spp. and *M. chelonae* are well-described agents of keratitis, other ameba-resistant organisms have not been associated with keratitis. However, 1 of the 2 new ameba-resistant bacteria isolated from the contact lens storage case liquid had a 16S rDNA sequence similar to that of the endosymbiont of an *Acanthamoeba* species isolated from the corneal sampling of a patient with keratitis in South Korea (*5*).

These data indicate the ubiquity of these emerging organisms and raise questions about roles of the ameba host and the symbiont as causative agents of keratitis. We therefore estimate that any of the organisms reported or any combination of these agents could have been involved in the keratitis that developed in the patient. Because the minute quantity of corneal scraping material prevented molecular detection, the relative contribution of each organism could not be specified.

This case illustrates that culturing ameba from ocular specimens and contact lens storage case liquid is mandatory for determining the diversity of pathogens potentially responsible for ameba-associated infections, such as keratitis, in patients who wear contact lenses. Ameba-resistant organisms have complex reciprocal interactions with the host, reminiscent of the mafia behavior.

The patient’s practice of not drying her hands after cleansing them with tap water and diluting the contact lens cleansing liquid with tap water may have provided a route for contamination of the contact lens fluid with ameba-resistant, tap water–borne organisms. Patients should be informed of the risk for keratitis caused by water-borne, amebal-resistant pathogens. They should also be educated to avoid contact with tap water when manipulating contact lenses, to dry hands after washing them with water and soap, or to use antiseptics containing >70% alcohol for hand disinfection before contact lens manipulation.
